# 

**Published:** 1864-05

**Authors:** 


*Contents.*

ORIGINAL COMMUNICATIONS........................................... 65

1. On the Microscopic Anatomy, Physiology and Pathology of the Human Liver, by Surgeon H. D. Schmidt. 2. On the Absence of Chlorides in the Urine of Persons affected with Variolous Diseases, by Surgeon J. C. M. Merillat. 3. On the Application of Smith's Anterior Splint, by Assistant-Surgeon Russell Murdock. 4. Resection of three-fourths of Clavicle for Osteo-Sarcoma, by Surgeon Charles Bell Gibson. 5. Case of Pericarditis and Double Pleurisy, with Effusion, reported by Surgeon E. S. Gaillard. 6. Case of Gun-Shot Wound of Kidney—Recovery—by Assistant-Surgeon R. J. Perry.

CONFEDERATE STATES HOSPITAL REPORTS.............. 75

Report of Wounded treated in Field Hospital of Hindman's Division, Army of Tennessee, after the battle of Chickamauga, by Chief Surgeon Carlyle Terry.

EDITORIAL AND MISCELLANEOUS.............................. 77

Amputation, Disarticulation and Resection Statistics of the Confederate States Army. The Prospect before Us.

CHRONICLE OF MEDICAL SCIENCE................................ 78

Comparative Results of Primary, Intermediate and Secondary Amputations, translated from the French of Legouest, by H. L. Thomas, M. D.

CONFEDERATE STATES

